# Revision of ICH S8 Needed?

**DOI:** 10.3389/ftox.2022.866737

**Published:** 2022-04-25

**Authors:** Jan Willem Van Der Laan

**Affiliations:** Senior Assessor Pharmacology and Toxicology, Medicines Evaluation Board, Utrecht, Netherlands

**Keywords:** carcinogenicity, immunotoxicity evaluation, nanopartical, immunostimulating activity, immunotoxicogenomics

## Introduction

The International Council of Harmonisation (ICH) Guideline S8 on Immunotoxicity Testing (ICH, 2005) started nearly 2 decades ago. In 2002 a meeting was being held in Brussels to discuss among different regulatory areas in the pharmaceutical world the need to request functional immunotoxicity for all human pharmaceuticals coming to the market, or for a selection only. At the end of the second millennium the European Union released a guideline indicating that for each new human pharmaceutical compound functional immunotoxicity should be tested, preferably by a T-cell Dependent Antibody Response testing (TDAR), or a test with a similar broad spectrum.

This approach was supported by the Japanese Pharmaceutical Manufacturers Association (JPMA), but the US Food & Drug Administration (FDA), Division on Immunological diseases, defended its position that in depth immunotoxicity testing should be requested only in case of concern. (for a review see Hastings, 2013).

The process of harmonisation started with gathering a dataset of chemicals including human pharmaceuticals, to find out whether important immunotoxic compounds would be missed if not tested specifically in this respect. Based on this dataset it became clear that in only 6 out of 45 cases (13%) the added functional testing of immunotoxicity did show sufficient evidence that these compounds were immunotoxic (Weaver et al., 2005).

So, the process finished with an agreement that a cause for concern approach instead of a general requirement of Additional Immunotoxicity Studies would be most appropriate. Six causes for concern have been identified: 1) findings from Standard Toxicity Studies, 2) the pharmacological properties of the drug, 3) the intended patient population, 4) structural similarities to known immunomodulators, 5) the disposition of the drug, and 6) clinical information, and it was decided that a single cause could be the reason to conduct more in depth studies with regard to immunotoxicity. The guideline finishes with an appendix describing what is meant by Additional Immunotoxicity Studies.

### Developments Since 2005

Is any update needed regarding this approach? Various new developments can be identified.

*Immunotoxicogenomics.* In a comparative study on mouse spleen with well-known immunotoxicants (Tributyltinoxide, Cyclosporin A, and Benz [a]Pyrene), paracetamol (acetaminophen) was clearly identified as a potential immunosuppressive agent (Baken et al., 2008), thus confirming findings published a few years earlier, i.e. the reduction of antibody responses in mice in the TDAR design (Ueno et al., 2000; Yamaura et al., 2002). The immunotoxic effect of paracetamol could not be detected in standard toxicity studies. Paracetamol influenced the gene expression in spleen lymphocytes consistent with inhibition of cell proliferation of immune cells (Baken et al., 2008). This immunosuppressive effect of paracetamol appeared to be relevant in humans as well, but mainly as an effect during an infectious challenge, with an association between use of paracetamol and increased duration of infection (Doran et al., 1989). This was confirmed in paediatric studies with pneumococcal vaccines (Prymula et al., 2009), i.e. a dose of paracetamol administered to prevent the vaccine-induced fever, decreased the immune response to the vaccine, whereas paracetamol had no effect on the immune response when already induced and the paracetamol was given on demand. Doedée et al. (2014) has confirmed this immunosuppressive effect of paracetamol with a similar design in human adults with Hepatitis B vaccination. The power of immunotoxicogenomic screening in mice has, therefore, been confirmed by these human relevant findings. It is recommended to include this methodology in a revised ICH S8 Guidance document.

*Immunotoxicity of nanoparticles.* Nanosized preparations were first used in humans in the 1930s, as colloidal intravenous iron-based products to treat iron-deficiency anaemia. Other nanomedicines were the liposomal products containing anti-cancer compounds such as doxorubicin, to be used as to target the distribution of these compounds (Ehmann et al., 2013). These authors describe also the novel block copolymer micelles as nanomedicines to achieve improved delivery of poorly soluble, highly toxic and/or unstable drugs, to increase tissue targeting and/or to improve the efficiency of cytosolic delivery of macromolecular drugs. Noorlander et al. (2015) offered an inventarisation of nanomedicines receiving a marketing authorisation since the start of the European Medicines Agency, and most of the real complex properties were identified as liposomal formulations or iron-sucrose particles, with a steady increase in the number of products. Monoclonal antibodies, although large nanosized molecules are completely different in their properties, and in my view do not deserve the classification of nanoparticles.

Dobrovolskaia et al. (2016) has reviewed the impact of nanoparticles on the immune stem, as these small particles are usually recognized as non-self in the mammalian body, and are characterized by Pathogen-Associated Molecular Patterns (PAMPs) at the outside surface of the particle. These PAMPs interact with receptors on immune cells named Pattern Recognition Receptors (PRRs). The recognition of these signals by PRRs leads to the activation of intracellular pathways in innate immune cells via Toll-like receptors located at the outer membranes of dendritic cells, which are specifically sensitive to this type of patterns. Engineering of nanoparticles can lead to either stimulatory or inhibitory properties with regard to the immune system. In a review paper Giannakou et al. (2020) has presented a proposal for adaptation of S8 Guideline with the following elements:

1. Nanospecific physicochemical properties, such as size and surface properties, which can specified as coating, chemistry/functionalisation, chemical structure, charge and hydrophobicity. Of course many of these properties are related to each other.
2. Intended use, which is focusing on its route of administration, i.e. intravenous for the majority of the products, but also topical or just dermal administration. In the latter case it is important whether penetration of the skin is purposed. If penetration through the skin is expected to be low, further study might not be needed, as immunotoxicity is only to be expected after systemic exposure. In such cases skin sensitization tests might be sufficient.
3. Endotoxin determination. For this type of products all must comply to limits of the endotoxin content, which is considered to be an impurity. The European Pharmacopoeia (2020) provides a threshold for this. Giannakou et al. (2020) has described a specific analytical method determining the presence of endotoxin-specific fatty acids, which can be helpful in this respect as the Limulus amoebocyte lysate test is not compatible with nanoparticle products. Strictly spoken this aspect is generally seen as a quality issue, and does not belong to the nonclinical safety part of development.
4. *In vitro* immunotoxicity testing for nanoparticles. The ICH S8 Guideline does not discuss specific *in vitro* tests for immunotoxicity evaluation of pharmaceuticals. Important areas are complement activation, macrophage function, inflammasome activation, myelosuppression, lymphocyte function, and dendritic cell antigen presentation. We refer to the paper of Giannakou et al., 2020 for more details on these aspects. For instance, an overblown complement activation response may lead to complement activation-related pseudoallergy (CARPA). Components of complement can be assayed in human serum with an ELISA, and when the complement cascade is being induced, no further testing is needed (Szebeni, 2014). The outcome of these *in vitro* studies can be regarded as predictive for the human situation.

A Weight-of-Evidence approach should be applied taking into consideration the results of the *in vitro* studies mentioned above in combination with the outcome of standard toxicity studies, along with the physicochemical properties. Such a WoE approach might be included in a revised ICH S8 Guidance.

*Immunosuppression and carcinogenicity.* One of the risks identified for immunosuppressants is carcinogenicity. This association is accepted as a common risk in humans for all immunosuppressants, and this is clearly identified in the ICH S8 Guideline. However, this is not true in rodents. Several papers indicate that less than 50% of all immunosuppressants are associated with cancer in rodent studies. Bugelski et al. (2010) has given an overview of 13 identified immunosuppressive agents (on the basis of increase risk of infection) in humans, indicating that from the 21 conducted rodent life-time studies in mice and rats only 33% has led to induction of immune-system related tumours, which makes the predictive value of rodent studies very low.

Lebrec et al. (2016) reports from a HESI-FDA-workshop held on this topic, and conclude that the cancer risk should be evaluated based on mechanism-based weight-of-evidence, including data from immune function tests related to tumour immunosurveillance.

Within the framework of the S1 Carcinogenicity topic in the process of the ICH there was a Prospective Evaluation Period (PEP), in which this issue was also on the table. Compounds for which sponsors could identify an outspoken immunosuppressive character could be placed in a category with an identified human risk, for which it was agreed that the conduct of a 2-years rat study would have no added value. Several case studies showed some immunotoxity, but the situation was not very clear to conclude about the full risk of immunosuppression which would obviate the need for a full carcinogenicity study. This will be discussed in a paper describing the dataset gathered in the PEP, which is now under preparation by the governmental members of the Expert Working Group. In this respect there seems to be no need to adapt the ICH S8 Guideline.

*Immunostimulation*. The focus of the ICH S8 Guideline is on immunosuppression as result of immunotoxicity, and already at the time of release there has been a discussion whether or not to include immunostimulation. In fact, the general background was just practicality: there was no disharmony among regulatory authorities how to handle immunostimulatory agents such as compound inducing sensitization. So, there was no need to invest precious money into negotiations on a harmonised issue.

The recommended approach in ICH S8 is to test the immune system in a functional way with the intention to take the system as a whole. The use of Sheep Red Blood Cells (SRBC) or Keyhole Limpet Haemocyanine (KLH) had the intention to stimulate the T-cell Dependent Antibody Response (TDAR), which is seen as a model assay of an integrated response of the immune system. A differentiation is possible between agents affecting the humoral system in contrast in favour above the cellular system, at least with respect to the immunostimulatory action (Bouteau et al., 2019). It is important to consider whether this differentiation between humoral and cellular responses is also possible with respect to the immunosuppressive action.

In addition, immunostimulation can be seen in different ways, i.e. as an intended effect or an unintended (toxic) effect such as hypersensitisation. As an intended effect it belongs to the area of the adjuvants which are included in vaccines to enhance the efficiency of the immune response protecting against infectious diseases (Bouteau et al., 2019). A regulatory guideline was written by the European Medicines Agency in 2004, but withdrawn recently in favour of the WHO Guideline on Adjuvanted Vaccines (2013). Although adjuvants are already for nearly 100 years now with the primary discovery of alum in 1926, only a few have reached the market during those years. Only the last 15 years there is more maturity with adjuvant-producing companies in developing adequate adjuvants, which became more apparent in the development of adjuvanted COVID-19 vaccines, such as Nuvaxovid (EPAR Nuvaxovid, 2021). Toxicity of adjuvants is likely to be related to their pro-inflammatory effect. Recently, Villeneuve et al. (2018) has expressed adverse outcome pathways, which might be helpful in identifying the biomarkers of inflammation in this respect.

Immunostimulation as inducing hypersensitivity was not yet included in the ICH S8, as at in 2006 predictive studies were only present for skin hypersensitisation. For systemic hypersensitisation neither *in vivo* nor *in vitro* assays have been identified. Systemic hypersensitisation is an important complication as an adverse effect. However, not until recently this was only identified in the first-in-human studies, as animal models were far from predictive. We refer to the paper from Iulini et al., 2022 (to be published) in this issue, and a multi-assay approach is recommended including a modified THP-1 activation assay, which enhances the identification of drugs with a high risk of inducing systemic hypersensitivity.

### Future Directions

*Extension of the scope?* The scope of S8 is limited to small molecules and therapeutic proteins are excluded. This also covers the large group of monoclonal antibodies which have by definition a direct connection to the immune system. Many monoclonal antibodies are on the market and others are still being developed. Why not all under the scope of ICH S8? Nonclinical immune-related safety assessments should cover all therapeutic modalities.

Is this criticism reasonable? Many monoclonal antibodies have indeed an immunological target, and a concern in this respect is easy to raise. However, we should not neglect the high specificity of therapeutic proteins and as a consequence the minimal risk to induce toxic effects (van Meer et al., 2013).

The latter aspect is also recognized in the ICH S6 Guideline, in which immunotoxicity (which is by definition different from immunogenicity) is also not identified as a specific topic. Important is the concept that the primary effects of therapeutic antibodies on the immune system will be studied *in extenso*, as this is needed to support its intended use. Conducting standardized functional assays such as the TDAR in nonhuman primates or even minipigs do not add value (Van Mierlo et al., 2014). Usually the pharmacodynamic data of these proteins are sufficient to decide about their safety too (Van der Laan et al., 2014).

*Role of T*_*regulatory*_ *-cells.* In the last decade, i.e. after release of the ICH S8 guideline, T_reg_ cells have received high interest. De Wolf et al. (2020) have strongly recommended to give more attention to the T_reg_, the T_regulatory_-cells. The authors evaluated 46 products, mainly monoclonal antibodies, using the registration dossiers submitted to the EMA. From these 46 a number of 7 products have been developed to target molecules with high relevance for T_reg_ function and survival. In addition, two EU-registered Janus kinase (JAK) inhibitors indicated for rheumatoid arthritis were included in this investigation.

The authors conclude that *Tregs have a crucial function in regulating immune responses to dampen inflammation, limit tissue damage and prevent autoreactivity. Pharmacological impact on their number and/or (local) activity, either directly or indirectly, is likely to contribute to (or impair) clinical responses or to adverse events. Therefore, monitoring effects of immunomodulatory products on Tcells -including Tregs-should be part of (pre-)clinical studies* (De Wolf et al., 2020).

There are many issues before Tregs can be used as biomarkers, as indicated by these authors, and further cooperation between industry, academia and governmental laboratories is needed to obtain more concrete regulatory guidance to include Tregs in monitoring in studies for marketing authorisation.

*Age-related effects on the immune system*. Further issues may include age-related effects of compounds on the immune system, e.g., in relation to paediatrics and use in elderly. The ICH S11 Guideline on Juvenile Toxicity Testing is finished recently and indicates that if a functional effect of a compound is identified in adult animals, there is no need to confirm this in juvenile animals (ICH 2021). Functional testing of the immune system is recommended not earlier than on PostNatalDay 45 in rats, as the immune system of very young animals is not appropriately developed for this purpose.

## Conclusion: Revision of ICH S8 Needed?

In this opinion paper we have reviewed a few scientific and regulatory issues related to immunotoxicology that have been brought forward in discussions whether or not to revise the ICH S8 Guideline on Immunotoxicity testing.

• The methodology of toxicogenomics is reaching the level of regulatory maturity, and immunotoxicogenomics is proven to be a good instrument to identify the human risks of certain products, which supports the inclusion of this methodology
• The field of nanoparticles is rapidly growing, and intended as well adverse effects on the immune system are being expected. Inclusion of this area in a revised guideline might have added regulatory value.
• One of the well-known risks of immunosuppression is an increase in human cancer. The evidence is growing that standard animal studies do not have added value in this respect. Deeper knowledge on the immunosuppressive properties might be sufficient to establish the carcinogenic potential.
• Immunostimulation, although not really excluded from the guideline, does not receive much attention either in the intended way, eg by using adjuvants in vaccines, or in the unintended way by inducing hypersensitisation. It is recommended to extend the area of S8 to include this area.
